# Evaluation of Patellar Tendon Structural Changes following Biological Treatments: Secondary Analysis of Double-Blinded Clinical Trial of Bone Marrow Mesenchymal Stromal Cells and Leukocyte-Poor Platelet-Rich Plasma

**DOI:** 10.3390/biomedicines12071599

**Published:** 2024-07-18

**Authors:** Silvia Ortega-Cebrián, Robert Soler-Rich, Lluis Orozco, Gil Rodas

**Affiliations:** 1 Physiotherapy Department, Facultat Fisioteràpia, Universitat Internacional de Catalunya (UIC), Carrer Josep Trueta, Sant Cugat de Vallès, 08017 Barcelona, Spain; 2 Medical Department of Football Club Barcelona (FIFA Medical Center of Excellence), Barça Innovation Hub of Football Club Barcelona, 08970 Barcelona, Spain; 3 Institut de Teràpia Regenerativa Tissular, Centro Médico Teknon, 08022 Barcelona, Spain; 4 Sports and Exercise Medicine Unit, Hospital Clinic and Sant Joan de Déu, 08029 Barcelona, Spain

**Keywords:** patellar tendon, imaging, tendinopathy

## Abstract

Objective quantification of tendon structural changes through imaging is only achieved by evaluating tendon structure using ultrasound tissue characterization (UTC) technology. This study compares the effects of bone marrow mesenchymal stromal cells (BM-MSC) and leukocyte-poor platelet-rich plasma (Lp-PRP) on tendon structure and clinical outcomes in male patients with patellar tendinopathy measured with UTC at 3, 6, and 12 months after treatment. This is a double-blinded clinical trial with a randomized active control study with 20 male patients diagnosed with patellar tendinopathy who underwent BM-MSC and Lp-PRP. Bilateral ultrasound tissue characterization scans of the patellar tendon were carried out after 3, 6, and 12 months, as well as tests for strength and pain. UTC patellar tendon was analyzed at the insertion, proximal, and mid-tendon. BM-MSC showed a greater capacity to promote further positive changes than Lp-PRP. Lp-PRP presented higher disorganized echo-type II in the mid-tendon (*p* = 0.04; ES = 1.06) and III (*p* = 0.02; ES = −1.47) after 3 months in the Lp-PRP group. Similar results were seen after 6 and 12 months. Pain and strength data show improvement in the treated tendon. BM-MSC treatment demonstrates a superior capacity to promote tendon regeneration and organization, restore strength, and reduce pain compared to Lp-PRP, after 3, 6, and 12 months in male patients with patellar tendinopathy.

## 1. Introduction

Patellar tendinopathy is characterized by pain, decreased strength, reduced functionality, and changes in tendon structure, primarily tendon disorganization [1]. Structural changes in the tendon are considered a major risk factor for developing this pathology. Disorganized tendon structure is identified through hypoechoic images associated with increased proteoglycans and water, creating space between tenocytes [2]. These spaces alter collagen’s adhesive capacity, compromising both the linear and parallel tendon structure, as well as the tendon’s mechanotransduction capacity and cellularity recovery [3]. Tendon structure changes are observed as alterations in the cross-sectional area (CSA), an increase in the tendon’s anteroposterior diameter, and discontinuity of collagen seen as proximal patellar tendon thickness [4]. Tendon thickening may occur as a compensatory response to ensure a higher number of parallel collagen bundles, maintaining the tendon’s role in storing and releasing energy [5,6,7,8].

Long-term excessive load can lead to impaired tendon recovery, resulting in tendon structural disorganization, dysfunction in force transmission, and pain [9,10]. Tendon dysfunction is reflected in biomechanical alterations; for example, individuals with patellar tendinopathy exhibit decreased strength and a stiff-knee landing mechanism. This stiff-knee landing is associated with deficits in energy absorption due to pain and possible tendon matrix alteration [6]. Pain may occur not only due to the presence of nociceptive substances in the tendon (e.g., acetylcholine, glutamine, substance P, catecholamines) resulting from neovascularization and neoinnervation [6,11] but also due to changes in pain modulation and sensitization as mechanisms of tissue protection and injury avoidance [7,12]. The explanation for chronic patellar tendon pain without clear evidence of injury remains vague. Despite a strong association between symptoms and tendon structure disorganization, changes in tendon structure have also been observed in asymptomatic tendons [5,13,14]. In a study, it was demonstrated changes in both symptomatic and contralateral asymptomatic tendons compared to a control group [14]. Mechanisms associated with contralateral tendon alteration include evasive biomechanical changes [15], central or systemic changes [11,12,16], the cross-education effect [17], and intrinsic factors such as genetic predisposition and adipose tissue [18]. Some authors suggest not using the asymptomatic contralateral tendon as a reference for comparison due to the apparent bilateral effect of tendon alterations [11,14,19].

Currently, quantification of tendon structural changes through imaging is primarily achieved by evaluating tendon matrix structure using ultrasound tissue characterization (UTC) technology. UTC algorithms quantify the stability of ultrasound image brightness across all 17 contiguous transverse images, classifying them into four echo-types (I–IV). UTC classifies tendon structure quality related to collagen adherence and organization from higher to lower quality [20]. Although UTC is not originally a diagnostic tool, the presence of echo-types III and IV seems to increase the risk of patellar tendon injury [21]. Docking et al. (2014) observed changes in tendon structure with UTC in both symptomatic and asymptomatic tendons; however, no long-term changes were demonstrated after therapeutic intervention despite the resolution of pain and functionality [15].

Management of patellar tendinopathy aims to decrease clinical symptoms and normalize tendon structure. In recent decades, there has been growing interest in normalizing tendon structure to improve mechanical properties and functionality. This has led to the development of biological treatments, mainly platelet-rich plasma (PRP) and stem cell therapy. PRP is considered safe and shows promise in promoting healing and reducing tendon injuries, although evidence for its effectiveness is still evolving, with some studies showing mixed results [22,23]. Stem cell therapy holds greater potential for tendon regeneration due to stem cells’ ability to differentiate into tendon tissue [24]. Research demonstrates positive outcomes, but larger, well-designed trials are needed for conclusive evidence [24]. There is a wide variety of biological treatments, but their ability to reverse tendon structure to organized fibers remains unclear. Currently, changes in symptomatic patellar tendon structure have been observed after 6 and 12 months of intervention with bone marrow mesenchymal stromal cells (BM-MSC) [25,26]. Goldberg et al., 2024, demonstrated positive clinical outcomes in mid-portion Achilles tendon with no significant structural changes after BM-MSC treatment [27]. Structural changes have been seen bilaterally, either related to treatment or load [14]. Although the existence of a bilateral factor (nociceptive, systemic, or biomechanical) in tendinopathy behavior is evident, it does not have a clear explanation. If changes in the tendon matrix structure occur in the asymptomatic contralateral tendon, either through evasive mechanisms or the cross-education effect, it could be questioned whether these mechanisms could occur in the process of tendon matrix normalization or reversing disorganized tendon fibers after a treatment. The bilateral cross-education effect has been demonstrated to increase muscle activation and strength by up to 8–11% from baseline in the untrained limb [17,28]. It is unclear whether there is a similar effect in tendon structures. 

In the literature, most evidence reporting improvement in tendon structure is based on measurement of tendon thickness and observational analysis of neovascularization. There is scare evidence describing the extent of tendon structure changes, whether towards an organized or disorganized tendon matrix, after biological treatment. 

The primary aim of this study is to compare the effects of bone marrow mesenchymal stromal cells (BM-MSC) and leukocyte-poor platelet-rich plasma (Lp-PRP) on tendon structure change in male patients with patellar tendinopathy measured with UTC after 12 months from treatment. The secondary aim is to compare patellar tendon structure differences between the two biological treatments after 3 and 6 months from treatment, as well as to assess structural changes between the symptomatic and asymptomatic tendon and clinical outcomes of quadriceps strength and the presence of pain at 3, 6, and 12 months after treatment.

The purpose of this study is to quantify the amount of change in tendon structure described by echo-types to determine which of the two biological treatments promotes more organized tendon fibers, greater strength, and reduced pain after 3, 6, and 12 months from treatment. The results could also secondarily contribute to the concept of the cross-education effect in the patellar tendon, supporting local management in symptomatic patellar tendinopathy or suggesting a contralateral approach in the asymptomatic tendon.

## 2. Materials and Methods

### 2.1. Design and Participants

This is a secondary analysis of a two-arm parallel-group, randomized, double-blinded controlled trial that involved male patients diagnosed with patellar tendinopathy who underwent cellular treatment with autologous expanded bone marrow mesenchymal stromal cells (BM-MSC) and leukocyte-poor platelet-rich plasma (Lp-PRP) at Institut de Teràpia Regenerativa Tissular (ITRT) at Centro Médico Teknon Hospital, Barcelona, Spain. Twenty male participants aged between 18 and 48 years were recruited. Male participants included in this study were aged between 18 and 48, had localized chronic pain in the patellar tendon for more than 4 months and palpation pain, with an intra-tendinous lesion >3 mm in length confirmed by T2 magnetic resonance imaging, presented no resolution of the pain after conservative rehabilitation treatment, and signed the informed written consent form. Participants with a grade III–IV osteochondral lesion, with anterior or posterior cruciate ligament injury, as well as participants who had received a local corticosteroid injection in the last 12 months, PRP injection in the last 6 months, had other pathologies or circumstances that may compromise participation in the study according to medical criteria were excluded from this study. All participants were recruited between December 2017 and November 2018 and monitored for 2 years following the protocol by Rodas et al., 2019 [29]. This single-center study was advertised to sports medicine physicians, orthopaedic surgeons, physical therapists, and the general public to recruit participants with patellar tendinopathy. The protocol was approved by the Ethics Committee for Clinical Research involving medicines at Quiron Hospital Group, Spain. This study was registered on ClinicalTrials.gov with reference NCT03454737 in December 2017 and EUDRA-CT number 2016-001262-28 and approved by the Spanish Agency of Medicines and Medical Devices (AEMPS).

Patients were randomized by the Clinical Research Organization (CRO) (Adknoma) using SAS statistical software (version 9.4) into either the BM-MSC group or Lp-PRP group in a 1:1 ratio. To keep patients and evaluating physicians blind to the treatment, sham procedures were performed in both groups (i.e., the BM-MSC group had blood extracted and had their tendon injected with normal saline at the first stage of treatment, and the Lp-PRP group underwent a sham bone marrow harvesting at the first stage of treatment). The treating physicians were not blind to the treatment administered. For this study, five groups were assigned: participants who received BM-MSC or Lp-PRP treatment were assigned as BM-MSC (n = 10) and Lp-PRP (n = 10). The untreated homologous contralateral tendon was assigned as asymptomatic BM-MSC (A-BM-MSC) (n = 10) and asymptomatic PRP (A-Lp-PRP) (n = 10). There were no losses or exclusions after randomization. The CONSORT reported flowchart diagram can be found in Rodas et al., 2021 [26].

In the original protocol, the authors decided that those participants who completed 6 months of Lp-PRP would be offered BM-MSC treatment, defined as phase B-BM-BMC. Consequently, for the purpose of this study, group five (B-BM-MSC) was considered as those participants that initially received Lp-PRP and after 6 months received BM-MSC. Hence, groups were assigned as BM-MSC, Lp-PRP, and B-BM-MSC for the symptomatic treated tendons, and A-BM-MSC, A-Lp-PRP, and A-B-BM-MSC for contralateral asymptomatic tendons which did not receive any treatment.

### 2.2. Procedure

Demographic data were collected before the study, and bilateral UTC scans of the patellar tendon were performed every month for 12 months. 

Data were collected before biological treatment (baseline), at three, six, and twelve months after the biological treatment. Isometric quadriceps strength and tendon pain were also collected. UTC scans were performed using B-mode ultrasound with a linear transducer of 7–10 MHz (SmartProbe 10L5; Terason 2000, Teratech, Rockville, MD, USA). An ultrasound probe (SmartProbe 12L5-V, Terason 2000+; Teratech) was fixed to a tracking device (UTC Tracker, UTC Imaging, Stein, The Netherlands) that automatically moves the transducer on the perpendicular axis of the tendon and records cross-images at 0.2 mm intervals [30]. 

Consistency of intensity and distribution of grey images was calculated over 4.8 mm using UTC algorithms. Four echo-types can be identified based on consistency, with echo-types I and II representing the organized and continuous tendon structure pattern, and echo-types III and IV the least organized tendon fibers. Echo-types are presented in percentage. Window size 17 was used for imaging analysis, the region of interest (ROI) was located around the tendon in the transverse view, and contours of the ROI were drawn at the patellar tendon insertion (10% tendon length), proximal tendon (20% tendon length), and mid-tendon (50% tendon length). The total tendon length is determined from the inferior angle of the patella to the most proximal tibia tuberosity. Tendon contours were marked by an experienced investigator (SOC), and echo-types were quantified through the UTC software itself (UTC 2010) [20]. Intra-rater reliability showed higher ICC values than inter-rater reliability; therefore, all UTC scans were conducted by one single tester (SOC) to decrease possible inter-tester biases. Using the same rater increased the reliability of UTC outcomes [30,31].

Pain levels were recorded using the visual analog scale (VAS) scores in each of the records. Quadriceps muscle strength testing was performed by maximum voluntary isometric contraction (MVIC) using a Mark-10 manual dynamometer, series 3 (Mark 10 Corporation, Copiague, NY, USA). Dynamometry was conducted on both lower limbs in a seated position with knee flexion at 90°. One end of the dynamometer was fixed to the examination table, and the force was applied to the other end through a strap placed around the distal third of the tibia. Three repetitions of 5 s each were performed with a 30 s rest after each repetition.

To achieve the primary aim of comparing the effects of bone marrow mesenchymal stromal cells (BM-MSC) and leukocyte-poor platelet-rich plasma (Lp-PRP) on tendon structure changes 12 months post-treatment, the primary outcome was defined as tendon structure changes evaluated using ultrasound tissue characterization (UTC) technology, specifically focusing on echo-type I. Secondary outcomes included echo-type changes at 3 and 6 months post-treatment, as well as echo-type changes in both the symptomatic and asymptomatic tendons, along with assessments of strength and pain levels

As this study is a secondary analysis of an RCT, the sample size was calculated based on the primary outcome of Rodas et al., 2021, where a sample size of 10 subjects per group was required to detect an effect size of 0.6 with 80% power for the effect of cellular treatments seen with MRI [26]. To assess whether 10 participants is an appropriate sample size for this study, we conducted a post-hoc power analysis using a power of 80% and a significance level of α = 0.05; 19 participants would have been required to demonstrate significant changes in echo-type I after 12 months (effect size = 3.48). 

### 2.3. Statistical Analysis

Changes in tendon structure were calculated for each echo-type at the insertion, proximal, and mid-tendon by the difference between the 3rd, 6th, and 12th months for BM-MSC and the 3rd and 6th months for Lp-PRP, with respect to baseline. Tendon structural difference for B-BM-MSC was calculated at the 3rd, 6th, and 12th months from phase B baseline. Phase B baseline was considered the timepoint, before receiving the B-BM-MSC treatment, being 6 months after receiving Lp-PRP treatment. Negative values for echo-types I and II mean greater tendon disorganization, while negative values for echo-types III and IV mean greater tendon organization.

**Figure 1:**
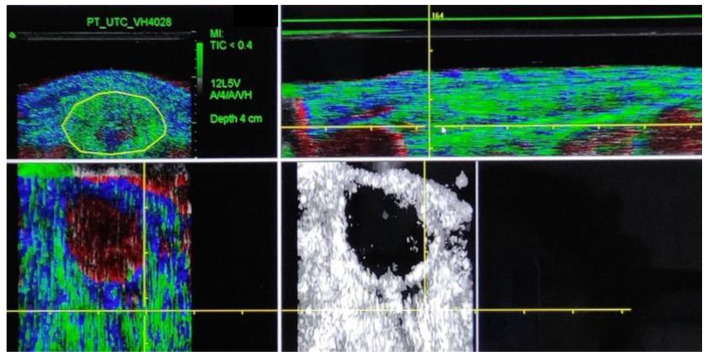
Ultrasound tissue characterization of the proximal tendon. Resection of the crossed sectional area at 20% tendon length. Color green, blue, red and black correspond to echo-type I, II, III, and IV respectively.

Descriptive statistics were calculated and examined for normality with the Shapiro–Wilk test showing normal distribution. Data are presented using mean and standard deviation (SD) for each echo-type. The intraclass correlation coefficient was calculated from our previous studies using the same methods as described above for the proximal and mid-tendon portion [32], see Figure 1. High reproducibility and moderate to excellent intra-observer reliability have been reported with UTC (ICC > 0.80) in trained observers [20,33]. To identify whether differences found were high enough to be evident, minimal detectable change (MDC) values for each echo-type were calculated. The standard error of measurement was calculated (SEM = standard deviation × √(1 − ICC), as well as MDC (MDC = 1.96 × SEM × √2).

To address the primary aim of this study, which is to identify tendon structure differences between biological treatments, we compared BM-MSC and Lp-PRP treatments. To identify tendon structure differences between BM-MSC treatments as the first treatment and the second treatment after receiving Lp-PRP, we compared BM-MSC and B-BM-MSC. Similarly, to identify differences in clinical outcomes of strength and pain, we performed the same comparison. 

To address the secondary aim of this study, which is to identify differences in tendon structure between the treated tendon and the homologous contralateral tendon, we compared BM-MSC and A-BM-MSC, as well as Lp-PRP and A-Lp-PRP.

For all comparisons, we used independent sample *t*-tests to calculate tendon structure differences after 3, 6, and 12 months at the insertion, proximal, and mid-tendon, as well as for strength and pain. Bonferroni corrections were applied to adjust for multiple testing for pain and strength.

## 3. Results

Since patients were recruited from a specialized clinic, all participants completed the data collection process between December 2017 and November 2018. Participants did not show statistical difference in age (mean ± SD) (BM-MSC 35.80 ± 10.03; Lp-PRP 32.00 ± 9.45) or body mass index (BM-MSC 25.75 ± 2.25; Lp-PRP 24.54 ± 1.9). Further characteristics of the participants are shown in the Appendix and Table 1 in Rodas et al., 2021 [26].

The percentage of tendon structural differences is presented in Appendix A as descriptive data: mean (SD), 95% confidence interval (CI) (superior limit to inferior limit), and MDC and effect size for each echo-type in the insertion, proximal, and mid-tendon areas at 3 and 6 months after Lp-PRP, and 3, 6, and 12 months after BM-MSC, and phase B-BM-MSC of the symptomatic and asymptomatic tendon. 

**Figure 2:**
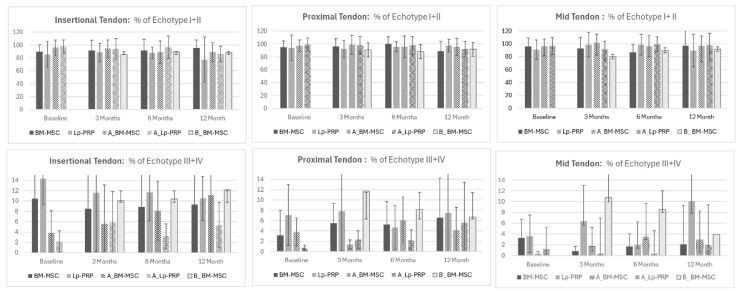
Tendon structural changes of echo-type I–IV at 3, 6, and 12 months after BM-MSC, Lp-PRP, and phase B-BM-MSC of the symptomatic and asymptomatic tendon at the insertional, proximal, and mid-tendon area (mean, 95% IC).

Mean percentage differences of organized tendons (echo-types I + II) and disorganized tendons (echo-types III + IV) at baseline, 3, 6, and 12 months after BM-MSC, Lp-PRP, and phase B-BM-MSC biological treatments are represented in Figure 2.

### 3.1. Evaluation of Echo-Types between BM-MSC and Lp-PRP

- Comparison between the differences in echo-types between BM-MSC and Lp-PRP showed higher disorganization in the Lp-PRP group after 3 months in echo-types II and III in the mid-tendon (echo-type II: BM-MSC 9.38 ± 14.81; MDC = 12.98; PRP −2.98 ± 8.4; MDC = 0.05; *p* = 0.04; ES = 1.06) (echo-type III: BM-MSC −0.65 ± 4.38; MDC = 4.85; PRP 9.61 ± 8.84; MDC = 0.29; *p* = 0.02; ES = −1.47).
- Similar results were seen after six months, with Lp-PRP still showing a higher disorganized tendon structure at echo-type III compared to BM-MSC (BM-MSC = −0.19 ± 2.01; MDC = 2.23; Lp-PRP 4.65 ± 5.17; MDC = 0.17; *p* = 0.03; ES = −1.4).
- Our results demonstrate that BM-MSC shows greater improvement in tendon organization than Lp-PRP at 3 and 6 months.

### 3.2. Evaluation of Echo-Types between BM-MSC and B-BM-MSC

- Comparisons of patellar tendon structure between BM-MSC as the first treatment and B-BM-MSC as the second biological treatment showed that BM-MSC presented more organized tendon structures at the proximal tendon for echo-type I after 6 months compared to B-BM-MSC (BM-MSC = 1.13 ± 12.6; MDC = 6.05; B-BM-MSC = −8.83 ± 20.46; MDC = 9.82; *p* = 0.04; ES = 0.58).
- Interestingly, long-term results showed more organized tendon fibers in the B-BM-MSC group compared to the first BM-MSC treatment for echo-types III and IV at the insertional tendon (echo-type III: BM-MSC = −1.67 ± 2.85; MDC = 2.62; B-BM-MSC 2.15 ± 6.18; MDC = 5.68; *p* = 0.04; ES = −0.79) (echo-type IV: BM-MSC = −0.89 ± 1.75; MDC = 2.02; B-BM-MSC 1.22 ± 3.32; MDC = 3.91; *p* = 0.04; ES = −0.79).

### 3.3. Evaluation of Clinical Outcomes between BM-MSC and Lp-PRP

- Clinical outcomes after 3 months of treatment showed A-BM-MSC and A-Lp-PRP to be significantly stronger compared to the affected tendon (BM-MSC; *p* = 0.01; ES = 3.92 and Lp-PRP; *p* = 0.02; ES = 4.36). At 6 months, A-Lp-PRP remained significantly stronger than Lp-PRP. Data for clinical outcomes show improvement in strength levels of the treated tendon and decreased pain levels. See Table 1.

### 3.4. Evaluation of Echo-Type between Symptomatic and Asymptomatic

- Patellar tendon structural changes between symptomatic and asymptomatic tendons showed minor significant differences. Only the mid-tendon showed significant differences for echo-type I in the BM-MSC group after 3 months. BM-MSC showed a higher amount of organized tendon fibers (6.92% ± 22.77) compared to A-BM-MSC (−2.35 ± 7.93; *p* = 0.02; ES = 0.1). Additionally, the MDC was 10.93% for BM-MSC and 3.8% for A-BM-MSC, with a large effect size of 0.1.
- The results confirm the positive effect of the treated tendon with BM-MSC compared to the untreated tendon. Conversely, Lp-PRP treatment did not show enough tendon structural change as the comparison between Lp-PRP and the asymptomatic Lp-PRP contralateral tendon showed no significant differences.

Table 2 presents a summary table of the statistically significant differences in patellar tendon structural differences of echo-types I–IV at the insertional, proximal, and mid-tendon areas of the symptomatic and asymptomatic tendons after 3, 6, and 12 months of receiving biological treatment. Complete numeric data of percentage structural tendon differences, including mean (SD), 95% IC, MDC, *p*-value, and effect size, can be seen in Appendix A. 

## 4. Discussion

Management of patellar tendinopathy focuses on decreasing pain, increasing functionality, and organizing and normalizing tendon fibrillar structure. Biological treatments have raised continuous growing interest in promoting cell regeneration and faster recovery in healthcare, as well as in tendinopathies. Management of tendinopathy with biological treatments shows promising results for stromal cell treatments compared to PRP. This study had the opportunity to examine short- and long-term patellar tendon structural changes between two biological treatments. This study provides quantitative information regarding patellar tendon structural changes between BM-MSC and Lp-PRP in the insertional, proximal, and mid-patellar tendon after 3, 6, and 12 months of biological treatment.

If changes in the tendon matrix structure occur in the symptomatic tendon after biological treatment, the contralateral asymptomatic tendon could present changes either through evasive mechanisms or a cross-education effect. This study investigated whether these mechanisms could occur in the process of tendon matrix normalization or reversing tendon disorganized fibers as a biological effect or a potential cross-education effect, as seen in muscle tissue.

The most remarkable results of this study were the positive effect of the BM-MSC treatment compared to Lp-PRP after 3 and 6 months at the insertional, proximal, and mid-tendon. In the short term, after 3 months of treatment, BM-MSC shows more organized fibers in echo-type II and fewer disorganized fibers in echo-type III than Lp-PRP in the mid-tendon. After 6 months, although the Lp-PRP group shows less mid-tendon disorganization, Lp-PRP presents higher disorganized echo-type III than the BM-MSC group.

Six months after biological treatment at the insertional tendon, both biological treatments show more organized fibers in echo-type I, although the BM-MSC group presents almost three times more organized echo-type I than Lp-PRP. BM-MSC showed a greater capacity to promote positive changes than Lp-PRP at the insertional, proximal, and mid-tendon. Tendon structural changes show a dynamic echo pattern for the BM-MSC group, indicating a positive difference in echo-type I, with a tendency for further organized tendon fibers compared to echo-type II. Despite the regulatory effects of mesenchymal stromal cells (MSC) not being clearly elucidated for patellar tendinopathy, the regenerative potential of mesenchymal cells has been explored with positive results [24]. Our results support progressively significant evidence of tendon tissue regeneration, previously reported by the improvement seen with MRI after 12 months in the BM-MSC group [25] compared to Lp-PRP. Mesenchymal cells are present in a wide range of tissues; bone marrow and adipose tissue are most frequently used for tendinopathy treatment, although further research is needed. According to our results, BM-MSC treatment shows a greater capacity to promote positive changes than Lp-PRP at the insertional, proximal, and mid-tendon after 6 months of treatment. These findings have also been reported in equine animals, supporting a greater tissue regeneration effect with BM-MSC compared to Lp-PRP and autologous bone marrow treatments (BM) [34]. Other clinical studies have compared the effects of PRP and adipose-derived mesenchymal stromal cells in different tissues, showing positive results, although further research is needed to clearly conclude long-term effects [35,36,37].

Further results from this study support the use of BM-MSC in the long term as the first-option treatment compared to Lp-PRP and phase B-BM-MSC. Our results also show a more positive response for the BM-MSC group as the first treatment than for participants who received BM-MSC after Lp-PRP treatment. Although differences are not seen after 3 months, more organized fibers (echo-type I) are seen in the mid-tendon after 6 months, and fewer disorganized tendon fibers are seen in the insertional tendon after 12 months in participants receiving BM-MSC as the first treatment.

These results confirm the positive effect of BM-MSC. The dynamic echo-pattern behavior of organized (echo-type I and II) and disorganized (echo-type III and IV) echo-types describes greater normalized tendon fibers and less disorganization after 12 months in the BM-MSC and B-BM-MSC groups compared to Lp-PRP. BM-MSC shows higher organized echo-type I and II in the long term, and phase B-BM-MSC shows higher organized echo-type III and IV, indicating positive organized tendon fibers in both treatments. Although biological treatments hold great promise in enhancing tissue regeneration, randomized controlled clinical trials and long follow-ups are needed to verify their full potential [22]. In Achilles tendinopathy, ultrasound imaging thickness decreased 24 weeks after BM-MSC treatment, although structural matrix changes were not seen in the six subjects who completed the follow up. Despite the lack of Achilles tendon structural changes, improvement in clinical outcomes was significant after BM-MSC [27].

In our study, clinical outcomes such as strength and pain demonstrated expected results, showing stronger legs than the asymptomatic leg after 3 months. Differences in pain and strength do not occur after 6 and 12 months for BM-MSC, but they still occur at 6 months for Lp-PRP. It seems that BM-MSC presents faster results in normalizing strength and pain compared to the asymptomatic leg than Lp-PRP and B-BM-MSC. Most of the evidence about the use of biological treatments shows similar effects in reducing pain and increasing strength mainly in the short term; further research is needed to confirm long-term effects [35,38]. These therapeutic interventions demonstrate clinically significant improvement, although minimal importance clinical difference (MCID) in pain and strength has not been found in the literature after receiving biological treatment. 

In tendinopathy, despite the strong association between symptoms and tendon structure disorganization, changes in tendon structure have also been observed in asymptomatic tendons [5,13,39]. Tendon structural changes are mainly related to load [40,41,42], and the potential cross-education effect on tendon structure has not been extensively studied. The bilateral crossed effect occurs mainly nociceptively [12], but could also be systemic [11]; however, explanations of the potential systemic effect remain scarce.

In this study, the only significant difference in tendon structure changes between BM-MSC and the asymptomatic BM-MSC occurs for echo-type I at the mid-tendon after 6 months. The amount of organized tendon fibers in the BM-MSC group is higher than in the asymptomatic BM-MSC group. Tendon structure after BM-MSC treatment tends to normalize tendon structure more than the contralateral tendons. Mid-term positive effects of BM-MSC compared to saline had already been shown in equine animals a decade ago [43,44]. The results demonstrate that while the BM-MSC cohort presents a tendency for further organized tendon fibers of echo-type I after 6 and 12 months, an echo-pattern of change is not seen in the asymptomatic tendon. We observe that greater fiber organization occurs at mid and long term, mainly in the proximal and mid-tendon.

In the Lp-PRP group, there are no structural tendon differences between symptomatic and asymptomatic Lp-PRP tendons. Our results show an initial negative effect in the Lp-PRP group, as organized tendon fibers seem to decrease in higher amounts mainly during the first 3 months. The short-term disorganized effect after Lp-PRP is reversed to a positive effect as the amount of degenerative tendon improves after 6 months. A similar echo-pattern is seen in the asymptomatic Lp-PRP group, showing a minor amount of change. Evidence of the effect of Lp-PRP is inconsistent and controversial due to the great variability of the preparation, localization, dose, and combination with further treatments such as medication and exercises. Negative effects and no effects are still often reported [23,45,46]. Our study supports the inconsistent effect of Lp-PRP on upregulating tendon cells after injections in the short and midterm. The effect of Lp-PRP treatment has shown positive effects compared to other non-invasive therapies [47]. Although the application of PRP has been related to stimulating fibroblast tenocyte proliferation and the production of collagen with organization [45], recommendations are to use it as a second-line treatment [38].

Although our study cannot demonstrate a contralateral cross-effect after biological treatment in the tendon, results show dynamic tendon adaptations in both tendons. Remodeling structural changes and clinical outcomes of pain and strength are enhanced after BM-MSC compared to Lp-PRP.

We acknowledge that the sample size is small, thus limiting the power of this study; due to the nature of this study, a sample size of 19 participants would have been appropriate to identify tendon structural changes after 12 months of treatment for echo-type I. We have considered limiting the inclusion of patients exclusively to males in order to reduce uncontrolled factors that could influence the results of this study. Clinical experience indicates that women respond more slowly to treatment. 

## 5. Conclusions

This current study supports the concept that the tendon adapts to compensate for areas of disorganization after biological treatment. It highlights the positive effects of BM-MSC treatment on tendon structure after 6 and 12 months. BM-MSC treatment shows a greater extent of organized and decreased disorganized patellar tendon structure, indicating the capacity to reverse misaligned tendon fibers. Lp-PRP shows less positive remodeling of structural tendon than BM-MSC after 3 and 6 months of treatment. In the midterm, the Lp-PRP results showed positive effects but were less significant than those of BM-MSC. The comparison of structural differences after receiving BM-MSC as the first treatment or after Lp-PRP treatment supports the use of BM-MSC as the first approach. Clinical outcomes of strength and pain normalized after biological treatment in the mid and long term. This study demonstrates dynamic changes in tendon structure in both the treated and contralateral tendons, ruling out a potential cross-education effect in tendons after biological treatment.

## Figures and Tables

**Table 1:** Clinical outcome, strength (Kg), and pain (VAS) of the symptomatic tendon after 3, 6, and 12 months after receiving biological treatment.

Descriptive Strength and Pain			N	Media	SD	CI		Effect Size	*p*-Value
						95% sup	95% inf		
Baseline	Strength	Lp-PRP	10	29.71	6.80	33.93	25.49	4.36	0.19
		A-Lp-PRP	9	31.44	3.81	6.18	3.15		
		BM-MSC	10	37.19	11.01	28.72	45.65	3.37	0.06 Ɨ
		A-BM-MSC	10	40.19	11.35	31.46	48.91	3.54	0.16 ƗƗ
	Pain	Lp-PRP	9	4.67	2.45	6.18	3.15	1.90	0.25
		BM-MSC	10	3.56	3.00	1.25	5.87	1.18	
3 Months	Strength	Lp-PRP	9	28.44	5.98	35.02	21.87	4.75	0.02
		A-Lp-PRP	8	31.44	9.41	37.27	25.61		
		BM-MSC	10	32.91	6.92	27.59	38.23	4.75	0.09 Ɨ
		A-BM-MSC	10	36.29	9.25	29.18	43.40	3.92	0.01 ƗƗ
		B-BM-MSC	9	34.83	14.04	43.53	26.12	0.85	0.07 ƗƗ
	Pain	Lp-PRP	10	1.50	2.01	2.75	0.25	0.74	0.07
		BM-MSC	10	2.00	3.04	−0.34	4.34		
		B-BM-MSC	8	1.12	2.23	2.43	−0.18	−0.26	0.56 ƗƗ
6 Months	Strength	Lp-PRP	8	28.91	5.23	35.61	22.21	5.53	0.03
		A-Lp-PRP	7	34.37	5.64	42.57	26.17		
		BM-MSC	10	33.37	9.25	26.26	40.48	3.60	0.55 Ɨ
		A-BM-MSC	10	34.24	9.33	27.07	41.41	3.67	0.41 ƗƗ
		B-BM-MSC	8	0.00	0.06	1.54	0.07	0.87	0.08 ƗƗ
	Pain	Lp-PRP	10	0.90	1.52	1.84	−0.04	0.59	0.17
		BM-MSC	10	1.89	3.06	−0.46	4.24		
		B-BM-MSC	8	1.51	1.04	1.21	−3.19	−0.70	0.13 ƗƗ
12 Months	Strength	Lp-PRP	9	33.15	9.69	39.15	27.15	3.42	0.87
		A-Lp-PRP	9	31.55	1.48	32.47	30.63		
		BM-MSC	10	38.43	9.72	30.96	45.90	3.95	0.51 Ɨ
		A-BM-MSC	10	40.31	11.33	31.61	49.02	3.55	0.09 ƗƗ
		B-BM-MSC	8	36.86	8.36	45.88	27.83	−0.17	0.71 ƗƗ
	Pain	Lp-PRP	9	1.22	2.54	−0.73	3.17	0.00	0.21
		BM-MSC	10	0.00	0.00	0.00	0.00		
		B-BM-MSC	9	1.13	1.40	14.67	−4.41	−0.62	0.18 ƗƗ

Ɨ Compared to Lp-PRP, ƗƗ compared to BM-MSC.

**Table 2:** Statistically significant differences in the patellar tendon structural differences of echo-types I–IV at the insertional, proximal, and mid-tendon of the symptomatic versus asymptomatic tendon after 3, 6, and 12 months of receiving biological treatment.

Months	BM-MSC and A-BM-MSC			Lp-PRP and A-Lp-PRP			BM-MSC and Lp-PRP			BM-MSC and B-BM-MSC		
	Insertional Tendon	Proximal Tendon	Mid-Tendon	Insertional Tendon	Proximal Tendon	Mid-Tendon	Insertional Tendon	Proximal Tendon	Mid-Tendon	Insertional Tendon	Proximal Tendon	Mid-Tendon
3			BM-MSC group show greater organization of echo-type I						Lp-PRP group show greater disorganization of echo-type II and III			
6									Lp-PRP group show greater disorganization of echo-type II and III		BM-MSC group show lower disorganized echo-type I	
12							Lp-PRP cohort show greater disorganization of echo-type I			BM-MSC group show lower disorganization of echo-type III and IV		

BM-MSC = Bone Marrow mesenchymal stem cells; Lp-PRP = leukocyte-poor Platelet-Rich Plasma; A-BM-MSC = Asymptomatic BM-MSC; A-Lp-PRP = Asymptomatic Lp-PRP; B-BM-MSC = phase B BM-MSC after Lp-PRP.

## Data Availability

CONSORT guidelines can be seen at supplementary data. The original contributions presented in the study are included in the article, further inquiries can be directed to the corresponding author.

## Supplementary Materials

The following supporting information can be downloaded at: https://www.mdpi.com/article/10.3390/biomedicines12071599/s1.

## Appendix A

**Table A1:** Percentage of tendon structural differences; mean (SD), 95% IC, MDC, *p*-value, effect size.

			BM-MSC			A-BM-MSC					Lp-PRP			A-Lp-PRP					BM-MSC-Lp-PRP		B-BM-MSC			BM-MSC-B-BM-MSC	
			Mean (SD)	95% CI (sup tp Inf)	MDC	Mean (SD)	95% CI (sup tp Inf)	MDC	*p*-Value	Cohens d	Mean (SD)	95% CI (sup tp Inf)	MDC	Mean (SD)	95% CI (sup tp Inf)	MDC	*p*-Value	Cohens d	*p*-Value	Cohens d	Mean (SD)	95% CI (sup tp Inf)	MDC	*p*-Value	Cohens d
3 months	Insertion	Type I	−12.26 (26.99)	(7.05 to −31.57)	12.96	−0.82 (10.45)	(6.65 to −8.29)	5.01	0.66	−0.26	−0.25 (13.82)	(12.45 to −12.93)	−0.26	0 (13.19)	(12.45 to −12.93)	−0.26	0.96	−0.01	0.23	−0.62	−15.06 (25.7)	−5.09 to −25.02)	12.33	0.85	0.1
		Type II	−4.16 (21.14)	(10.96 to −19.28)	18.53	−0.9 (8.71)	(5.32 to −7.14)	7.63	0.3	−0.15	−3.41 (10.31)	(4.56 to −17)	−0.15	2.81 (12.52)	(4.59 to −17.03)	−0.15	0.24	−0.12	0.92	−0.04	−5.29 (17.57)	1.51 to −12.11)	15.4	0.92	0.05
		Type III	−1.98 (9.58)	(4.87 to −8.83)	10.62	1.53 (3.83)	(4.27 to −1.21)	4.25	0.18	0.09	2.52 (5.1)	(11.33 to −0.91)	0.09	−2.69 (7.66)	(11.39 to −0.97)	0.09	0.09	0.13	0.21	−0.64	−0.08 (11.06)	4.2 to −4.37)	12.26	0.65	−0.18
		Type IV	−1.67 (4.04)	(1.22 to −4.56)	3.54	0.19 (0.64)	(0.64 to −0.26)	0.56	0.23	−0.05	1.13 (2.13)	(4.82 to −2.44)	−0.05	−0.05 (5.03)	(4.95 to −2.57)	−0.05	0.5	0.23	0.07	−0.94	−0.53 (5.71)	1.68 to −2.74)	5	0.54	−0.23
	Proximal Tendon	Type I	1.43 (17.96)	(14.28 to −11.42)	8.62	−7.16 (12.84)	(2.02 to −16.34)	6.16	0.87	−0.23	−12.91 (25.89)	(13.08 to −32.19)	−0.23	−3.35 (20.12)	(12.81 to −31.92)	−0.23	0.37	−0.4	0.16	0.91	−6.52 (21.53)	1.82 to −14.87)	10.33	0.33	0.4
		Type II	0.76 (11.44)	(8.94 to −7.42)	10.02	−0.12 (12.59)	(8.89 to −9.13)	11.04	0.27	0.02	−7.45 (21.69)	(2 to −30.88)	0.02	6.98 (9.05)	(1.84 to −30.72)	0.02	0.07	−0.15	0.3	0.85	0.68 (11.88)	5.29 to −3.91)	10.41	0.98	0
		Type III	−0.3 (9.85)	(6.74 to −7.34)	10.92	4.18 (7.85)	(9.79 to −1.43)	8.7	0.14	0.25	0.19 (2.69)	(13.19 to −7.34)	0.25	−2.73 (15.17)	(14.64 to −8.79)	0.25	0.58	−0.11	0.88	−0.07	0.05 (10.57)	4.15 to −4.04)	11.72	0.82	−0.03
		Type IV	−1.3 (4.64)	(2.02 to −4.62)	4.07	3.09 (7.62)	(8.54 to −2.36)	6.68	0.08	0.06	0.19 (1.5)	(6.89 to −4.07)	0.06	−1.22 (8.09)	(7.66 to −4.84)	0.06	0.61	−0.07	0.35	−0.45	−1.06 (4.85)	0.81 to −2.94)	4.25	0.94	−0.05
	Mid−tendon	Type I	6.92 (22.77)	(23.21 to −9.37)	10.93	−2.35 (7.93)	(3.32 to −8.02)	3.8	0.02 *	0.1	−8.88 (9.43)	(15.02 to −20.66)	0.1	−6.06 (15.35)	(29.77 to −35.41)	0.1	0.78	−0.69	0.07	0.85	−0.67 (21.86)	7.8 to −9.15)	10.49	0.39	0.33
		Type II	9.38 (14.81)	(19.97 to −1.21)	12.98	−4.12 (8.02)	(1.61 to −9.85)	7.03	0.05	0.05	−2.98 (8.4)	(9.2 to −33.7)	0.05	9.26 (22.7)	(40.22 to −64.72)	0.05	0.45	0.08	0.04 *	1.06	6.05 (13.21)	11.17 to 0.92)	11.58	0.41	0.23
		Type III	−0.65 (4.38)	(2.48 to −3.78)	4.85	4.64 (6.81)	(9.51 to −0.23)	7.56	0.46	0.26	9.61 (8.84)	(26.95 to −1.05)	0.26	−3.33 (8.65)	(29.74 to −3.85)	0.26	0.09	0.6	0.0 2 *	−1.47	0.25 (9.87)	4.07 to −3.57)	10.94	0.94	−0.12
		Type IV	1.02 (5.94)	(5.27 to −3.23)	5.2	2.67 (3.54)	(5.2 to 0.13)	3.1	0.23	0.46	3.71 (3.73)	(13.07 to −1.77)	0.46	−1.93 (6.72)	(20.22 to −8.92)	0.46	0.27	0.38	0.26	−0.52	0.45 (5.67)	2.65 to −1.74)	4.97	0.63	0.09
6 months *	Insertion	Type I	−11.42 (28.18)	(8.74 to −31.58)	13.53	2.99 (8.95)	(9.39 to −3.41)	4.29	0.4	−0.03	−0.19 (9.71)	(16.53 to −6.47)	−0.03	−5.23 (14.33)	(16.65 to −6.59)	−0.03	0.37	−0.16	0.25	−0.56	−17.08 (28.55)	−6.01 to −28.15)	13.7	0.46	0.19
		Type II	−6.55 (17.06)	(5.65 to −18.75)	14.95	−1.29 (9.21)	(5.29 to −7.87)	8.07	0.93	−0.26	−0.99 (9.3)	(6.62 to −15.78)	−0.26	3.59 (14.05)	(6.74 to −15.9)	−0.26	0.4	0.04	0.38	−0.46	−6.15 (16.33)	0.18 to −12.48)	14.32	0.59	−0.02
		Type III	−1.18 (4.71)	(2.18 to −4.54)	5.22	−1.05 (2.24)	(0.55 to −2.65)	2.48	0.9	−0.35	0.71 (1.36)	(3.62 to −3.02)	−0.35	0.41 (4.81)	(3.8 to −3.2)	−0.35	0.85	0.24	0.24	−0.56	1.33 (6.48)	3.85 to −1.18)	7.19	0.21	−0.44
		Type IV	−0.66 (2.56)	(1.17 to −2.49)	2.24	−0.77 (1.46)	(0.28 to −1.82)	1.28	0.25	−0.39	0.48 (0.97)	(1.63 to −3.19)	−0.39	1.26 (3.5)	(1.77 to −3.33)	−0.39	0.51	0.4	0.21	−0.61	0.61 (2.78)	1.69 to −0.46)	2.44	0.09	−0.47
	Proximal Tendon	Type I	1.13 (12.6)	(10.14 to −7.88)	6.05	−5.02 (10.73)	(2.65 to −12.69)	5.15	0.46	−0.18	1.07 (8.73)	(19.26 to −9.95)	−0.18	−3.57 (17.5)	(19.03 to −9.72)	−0.18	0.49	−0.04	0.99	0	−8.83 (20.46)	−0.9 to −16.77)	9.82	0.04 *	0.58
		Type II	−0.37 (8.5)	(5.71 to −6.46)	7.45	3.21 (12.69)	(12.29 to −5.87)	11.12	0.56	0.1	−2.78 (9.11)	(2.02 to −19.13)	0.1	5.76 (11.08)	(1.9 to −19)	0.1	0.1	0	0.57	0.36	1.02 (13.12)	6.11 to −4.06)	11.5	0.4	−0.12
		Type III	−0.2 (3.75)	(2.48 to −2.88)	4.16	0.69 (3.04)	(2.86 to −1.48)	3.37	0.37	0.08	1.11 (1.91)	(9.16 to −3.18)	0.08	−1.87 (7.96)	(9.18 to −3.2)	0.08	0.3	0.02	0.35	−0.45	0.73 (5.34)	2.8 to −1.33)	5.92	0.47	−0.2
		Type IV	−0.19 (1.66)	(1 to −1.38)	1.45	1.11 (4.13)	(4.06 to −1.84)	3.62	0.13	0.07	0.63 (1.01)	(4.24 to −2.39)	0.07	−0.28 (4.28)	(4.25 to −2.4)	0.07	0.54	0.15	0.21	−0.62	0.18 (2.28)	1.07 to −0.7)	2	0.47	−0.18
	Mid−tendon	Type I	6.54 (17.94)	(19.37 to −6.29)	8.61	−5.84 (11.39)	(2.31 to −13.99)	5.47	0.14	−0.07	−17.63 (19.94)	(25.65 to −44.42)	−0.07	−8.25 (13.08)	(33.66 to −52.43)	−0.07	0.49	−0.82	0.01 *	1.35	−2.11 (17.19)	4.55 to −8.78)	8.25	0.05	0.49
		Type II	11.26 (17.95)	(24.1 to −1.58)	15.74	1.15 (9.8)	(8.16 to −5.86)	8.59	0.19	0.37	−1.08 (13.01)	(12.23 to −31.54)	0.37	8.56 (18.07)	(27.95 to −47.26)	0.37	0.46	0.14	0.11	0.91	6.66 (14.4)	12.25 to 1.08)	12.62	0.48	0.28
		Type III	−0.19 (2.01)	(1.24 to −1.62)	2.23	1.4 (3.16)	(3.66 to −0.86)	3.5	0.84	0.17	4.65 (5.17)	(19.37 to −3.85)	0.17	−3.11 (12.84)	(37.37 to −21.85)	0.17	0.4	0.26	0.03 *	−1.41	1.18 (4.66)	2.99 to −0.62)	5.17	0.31	−0.38
		Type IV	0.47 (2.86)	(2.51 to −1.57)	2.51	0.26 (1.73)	(1.5 to −0.98)	1.52	0.15	0.15	1.57 (2.01)	(2.89 to −3.28)	0.15	1.76 (2.04)	(3.8 to −4.19)	0.15	0.89	0.81	0.35	−0.46	0.8 (2.34)	1.71 to −0.11)	2.05	0.67	−0.12
12 months	Insertion	Type I	11.31 (9.94)	(3.55 to −39.29)	8.75	2.18 (8.54)	(8.28 to −3.92)	8.2	0.89	−0.17	3.29 (9.21)	(12.16 to −10.18)	−0.17	2.3 (14.07)	(12.29 to −10.31)	−0.17	0.85	0.27	0.04 *	−0.99	−21.77 (30.56)	−9.92 to −33.63)	29.34	0.81	0.12
		Type II	−4.28 (16.76)	(7.71 to −16.27)	20.78	−3.5 (7.61)	(1.94 to −8.94)	9.44	0.06	−0.35	−2.44 (10.39)	(11.53 to −6.25)	−0.35	−5.08 (8.43)	(11.55 to −6.27)	−0.35	0.54	−0.34	0.77	−0.15	−4.43 (16.35)	1.9 to −10.77)	20.27	0.93	0
		Type III	−1.67 (2.85)	(0.36 to −3.7)	2.62	0.85 (2.95)	(2.96 to −1.26)	2.71	0.06	−0.14	−0.67 (2.56)	(2.65 to −6.27)	−0.14	1.14 (6.21)	(2.82 to −6.44)	−0.14	0.41	−0.01	0.42	−0.48	2.15 (6.18)	4.55 to −0.24)	5.68	0.04 *	−0.79
		Type IV	−0.89 (1.72)	(0.34 to −2.12)	2.02	0.5 (1.45)	(1.54 to −0.54)	1.71	0.62	−0.08	−0.19 (0.97)	(1.22 to −4.88)	−0.08	1.64 (4.5)	(1.42 to −5.08)	−0.08	0.23	0.19	0.28	−0.57	1.22 (3.32)	2.51 to −0.06)	3.91	0.04 *	−0.79
	Proximal Tendon	Type I	−0.17 (15.16)	(10.66 to −11.02)	14.55	−3.17 (11.02)	(4.71 to −11.05)	10.58	0.83	−0.14	2.8 (6.79)	(17.25 to −10.25)	−0.14	−0.7 (17.03)	(17.11 to −10.11)	−0.14	0.58	0.15	0.58	−0.25	−12.61 (23.96)	−3.31 to −21.9)	23.01	0.05	0.61
		Type II	0.93 (12.27)	(9.71 to −7.85)	15.22	−0.13 (10.47)	(7.36 to −7.62)	12.98	0.13	0.03	−1.82 (5.88)	(4.93 to −14.8)	0.03 *	3.11 (11.82)	(4.77 to −14.64)	0.03	0.28	−0.02	0.54	0.29	1.7 (13.07)	6.77 to −3.37)	16.2	0.69	−0.06
		Type III	−0.99 (3.47)	(1.49 to −3.47)	3.19	1.45 (3.58)	(4.01 to −1.11)	3.29	0.17	0.05	−0.73 (2.97)	(5.01 to −3.77)	0.05	−1.35 (5.09)	(4.93 to −3.69)	0.05	0.76	−0.25	0.87	−0.09	2.53 (8.39)	5.79 to −0.71)	7.71	0.13	−0.54
		Type IV	−0.58 (1.83)	(0.72 to −1.88)	2.15	1.83 (4.96)	(5.37 to −1.71)	5.83	0.16	0.02	−0.25 (1.05)	(2.52 to −2.49)	0.02 *	−0.26 (3.16)	(2.51 to −2.48)	0.02	0.98	−0.14	0.63	−0.23	1.09 (3.64)	2.5 to −0.31)	4.28	0.11	−0.57
	Mid−tendon	Type I	7.53 (26.82)	(26.71 to −11.65)	25.75	−6.73 (8.1)	(−0.92 to −12.53)	7.78	0.33	−0.27	−0.9 (7.47)	(21.47 to −4.77)	−0.27	−9.25 (0.63)	(15.27 to 1.42)	−0.27	0.02	−0.7	0.36	0.4	−0.93 (19.68)	6.69 to −8.56)	18.9	0.16	0.35
		Type II	8.12 (16.8)	(20.13 to −3.89)	20.82	1.88 (10.51)	(9.4 to −5.64)	13.03	0.14	0.33	0.48 (6.65)	(23.34 to −12.17)	0.33	−5.1 (18.66)	(150.97 to −139.8)	0.33	0.74	−0.12	0.21	0.58	3.84 (14.03)	9.28 to −1.59)	17.4	0.43	0.27
		Type III	−0.44 (0.84)	(0.16 to −1.04)	0.78	1.65 (4.08)	(4.57 to −1.27)	3.75	0.55	−0.05	0.27 (4.07)	(2.81 to −17.47)	−0.05	7.6 (10.04)	(67.92 to −82.58)	−0.05	0.48	0.44	0.66	−1.04	1.52 (4.78)	3.37 to −0.32)	4.39	0.11	−0.57
		Type IV	0.45 (1.08)	(1.22 to −0.32)	1.27	1 (2.66)	(2.9 to −0.9)	3.13	0.06	0.39	0.1 (1.41)	(0.43 to −13.73)	0.39	6.75 (9.26)	(74.3 to −87.6)	0.39	0.49	0.57	0.59	0.41	1.32 (3.48)	2.67 to −0.02)	4.09	0.47	−0.33

SD = standard deviation; CI = coefficient interval; Sup Lim = superior limit; Inf Lim = inferior limit; MCD = minimal detectable change; * = *p* value < 0.005.
